# Supplementary figures and images for: Clinical outcomes and mortality in old and very old patients undergoing cardiac resynchronization therapy
Source: PLoS One. 2019 Dec 2;14(12):e0225612. doi: 10.1371/journal.pone.0225612 (PMC6886771; doi:10.1371/journal.pone.0225612)

**Figure I - Cardiovascular survival in the 3 groups**

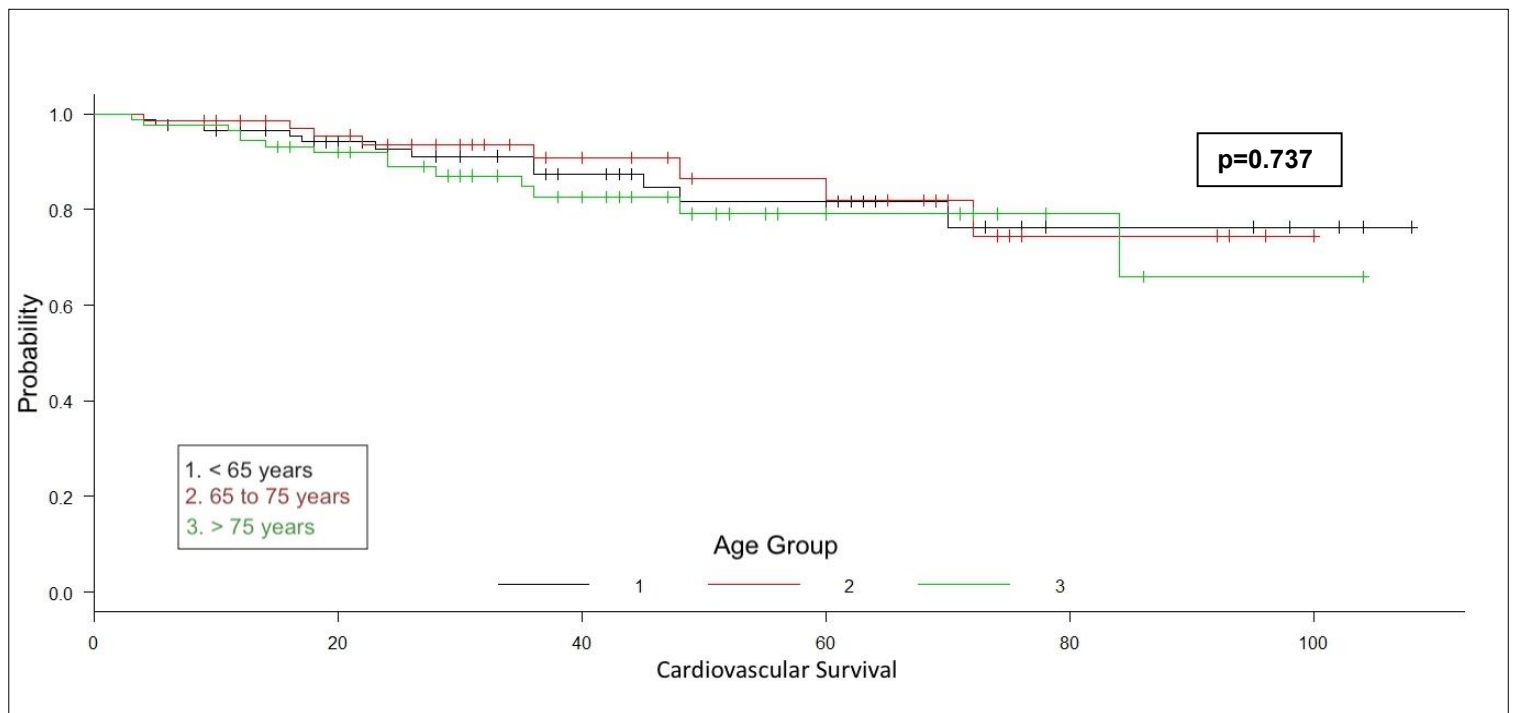

Supplement: S1 Fig — (PDF) [file pone.0225612.s001.pdf]

Figure II - Total survival in the 3 groups

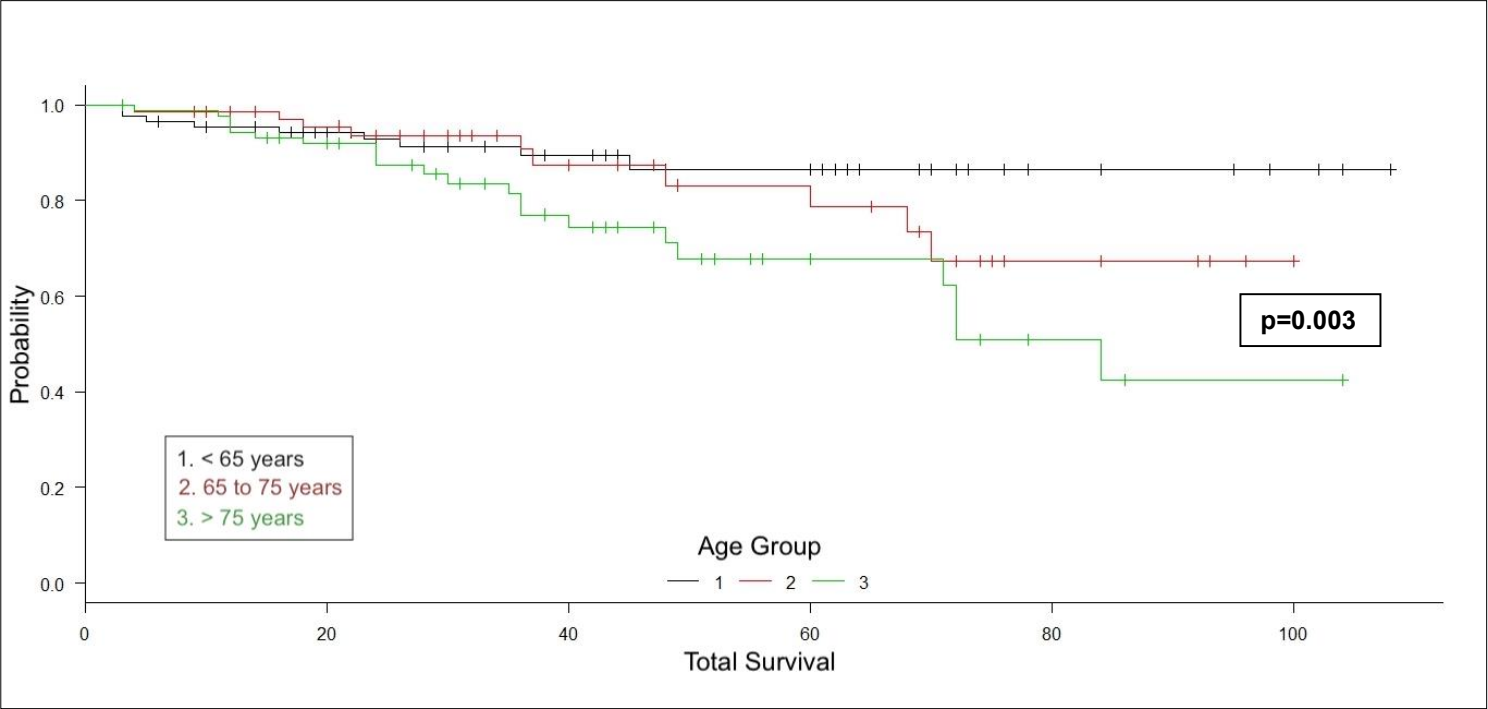

Supplement: S2 Fig — (PDF) [file pone.0225612.s002.pdf]
