# Supplementary material for: Clinical outcomes and mortality in old and very old patients undergoing cardiac resynchronization therapy
Source: PLoS One. 2019 Dec 2;14(12):e0225612. doi: 10.1371/journal.pone.0225612 (PMC6886771; doi:10.1371/journal.pone.0225612)
Supplement: Attachment — Submitted filename: Response to Reviewers.docx [file pone.0225612.s003.docx]

**Dear Academic Editor and Reviewers**:

Below is the answer to the comments of the reviewers:

1. **Reviewer # 1**:

**“*In conclusion, given the overall work, I accept manuscript with minor revision concerning a small implementation of bibliography, reporting the aforementioned works and other”.***

The implementation of bibliography was made and the articles were inserted in the *Discussion* (number 25 and 27) – pages 14,15 and 20,21.

*Although comparative studies do not demonstrate a direct correlation between clinical response and echocardiographic response, when a reverse remodeling criterion is associated with CRT assessment, the non-response rate was described to range from 35% to 40% [24,25]. Our results reflect this situation and the need for a more definitive classification of the problem, as when using only the FC, the response rate to CRT in the 3 groups was 82.8%, 94.9%, and 91%, respectively. When an echocardiographic reverse remodeling criterion was included, the response rate was 52.3%, 56.9%, and 68.5%, respectively.*

*Although there are >3500 scientific publications on ICD/CRT device implantation in patients aged >65 years, there are around 20 publications about the safety of the procedure in patients aged >75 years [26,27].*

25. Caritá P, Corrado E, Pontone G, Curnis A, Bontempi L, Novo G et al. Non-responders to cardiac resynchronization therapy: Insights from multimodality imaging and electrocadiography. A brief review. *International Journal of Cardiology*, 225,402-407.

27.	Adelstein EC, Liu J, Jain S, Schwartzman D, Althouse AD, Wang NC, et al. Clinical Outcomes in cardiac resynchronization therapy-defibrilaltor recipients 80 years of age and older. *Europace*, 18(3), 420-427.

2. **Reviewer # 2**:

***“There are 3 important data to explain”***

***- “The difference in ischemic and non ischemic population in the third Group”***

This paragraph below was inserted in *Results/ Primary endpoint: cardiac resynchronization therapy response and mortality (page 11)*.

In this group (>75 years), ischemic etiology, defined as previous myocardial infarction, PTCA, myocardial revascularization or severe coronary obstructive disease, was observed in 53.9%. No clinical differences and outcomes were observed between ischemic and non-ischemic very old patients.

**- *“The difference in mortality between Crtp and Crtd”***

This paragraph below was inserted in *Results/ Primary endpoint: cardiac resynchronization therapy response and mortality (page 11)*.

CRT-D was the prosthesis used in 75% of the patients approximately. When survival curves were analyzed according to the type of prosthesis, it was observed that CRT-p or CRT-d were not predictors of cardiovascular or total mortality, with similar survival rates.

**- *“The difference in response between population with LBBB and other wide QRS”***

This paragraph below was inserted in *Results/ Primary endpoint: cardiac resynchronization therapy response and mortality (page 11)*.

The LBBB occurred in 93%, 94% and 98%, respectively, in the three groups. In 12/ 249 pt (4.8%) was observed right bundle branch block (RBBB), 6 in group I, 2 in group II and 4 in group III. The QRS width average was 167 ms. Except one patient in group I, all RBBB pt were responders to CRT.
